# Sarcopenia risk in women with postmenopausal osteoporosis: a SARC-F-based assessment

**DOI:** 10.55730/1300-0144.6352

**Published:** 2026-05-06

**Authors:** Burak Kamil TURAN, Ayşe A. KÜÇÜKDEVECİ, Zafer GÜNENDİ, Ayşegül YAMAN, Oya ÖZDEMİR, Jale MERAY, F. Yeşim GÖKÇE KUTSAL

**Affiliations:** 1 Department of Physical Medicine and Rehabilitation, Faculty of Medicine, Ankara University, Ankara, Turkiye; 2 Department of Physical Medicine and Rehabilitation, Faculty of Medicine, Gazi University, Ankara, Turkiye; 3 Department of Physical Medicine and Rehabilitation, Ankara Gülhane Health Application and Research Center, University of Health Sciences, Ankara, Turkiye; 4 Physical Medicine and Rehabilitation Hospital, Ankara Etlik City Hospital, Ankara, Turkiye; 5 Department of Physical Medicine and Rehabilitation, Faculty of Medicine, Hacettepe University, Ankara, Turkiye

**Keywords:** Muscle strength, postmenopausal osteoporosis, physical activity, probable sarcopenia, sarcopenia risk

## Abstract

**Background/aim:**

Osteoporosis and sarcopenia are diseases of bone and muscle tissue, respectively. Studies reported that these disorders were interrelated. However, this relation was explored in older adults and/or with certain comorbidities, and information about nonelderly postmenopausal women is scarce. This study’s objectives were to investigate the relationship between osteoporosis and SARC-F-defined sarcopenia risk in postmenopausal women aged ≤70 years and to examine the effect of other factors on this risk.

**Materials and methods:**

This multicenter case-control study included postmenopausal women aged 45–70 years. Dual-energy X-ray absorptiometry was utilized to diagnose osteoporosis. Sociodemographic and clinical variables, sarcopenia risk (SARC-F), muscle strength (grip strength and five-times sit-to-stand test: 5T-STS), and physical activity levels (International Physical Activity Questionnaire-Short Form) were compared between osteoporotic and nonosteoporotic participants.

**Results:**

A total of 119 osteoporotic (median age: 58 years) and 119 nonosteoporotic (median age: 59 years) participants were recruited. No differences were observed between the osteoporotic and nonosteoporotic groups regarding SARC-F-defined sarcopenia risk (48.7% vs. 38.7%, p = 0.117), probable sarcopenia (14.5% vs. 15.3%, p = 0.876), grip strength (23 vs. 22 kg, p = 0.329), 5T-STS (13.90 vs. 13.96 s, p = 0.986), or physical activity levels (907.5 vs. 792 MET-min/week, p = 0.983). Although educational level [odds ratio (OR): 0.366, 95% confidence interval (CI): 0.189–0.707, p = 0.003], proton pump inhibitor (PPI) use (OR: 2.494, 95% CI: 1.227–5.068, p = 0.012), and physical activity level (OR: 0.966, 95% CI: 0.945–0.988, p = 0.003) were independent predictors of SARC-F-defined sarcopenia risk, osteoporosis was not (p = 0.176).

**Conclusion:**

SARC-F-defined sarcopenia risk was not increased in women with postmenopausal osteoporosis aged ≤70 years compared to nonosteoporotic controls. Lower educational status, PPI use, and low physical activity levels were associated with an increased SARC-F-defined sarcopenia risk.

## 1. Introduction

Osteoporosis and sarcopenia are diseases of bone and muscle tissue, respectively. Osteoporosis is characterized by low bone mass and microarchitectural deterioration of bone tissue [1]. Sarcopenia is a disease of skeletal muscle defined by low muscular strength and low muscle quality or quantity [2]. Both are systemic diseases related to fractures, physical disability, and mortality [2,3]. They particularly affect elderly people, and their prevalence increases with advancing age [4–6].

There is an interaction between bone and muscle tissues, and they regulate each other’s functions. This is facilitated by mechanical, paracrine, and endocrine signals. Therefore, it is possible that osteoporosis and sarcopenia are related diseases with similar origins [7]. In a pooled analysis comprising studies mostly focused on older adults or individuals with particular medical conditions, osteoporosis and sarcopenia were found to be highly correlated [8], and osteoporosis was reported as a risk factor for sarcopenia in older adults [4]. In older postmenopausal women, a strong relationship between osteoporosis and sarcopenia was also revealed [9], and the risk of osteoporosis escalated with the increasing severity of sarcopenia [10]. However, the studies in the literature investigating the relationship between osteoporosis and sarcopenia have mainly focused on the elderly population. Information on nonelderly postmenopausal women is scarce. Given these facts, the primary aim of this study was to investigate the relationship between osteoporosis and SARC-F-defined sarcopenia risk in postmenopausal women aged 70 years and younger, with a focus on screening-level assessment. The secondary aim was to examine the effect of other factors, including age, body mass index (BMI), clinical risk factors for osteoporosis, medication use, protein intake, and physical activity level, on SARC-F-defined sarcopenia risk in that group.

## 2. Materials and methods

This study was ethically approved by the relevant ethics committee on 13 January 2020. Written informed consent was obtained from all individuals before enrollment. The study was performed in compliance with the Declaration of Helsinki. The study was conducted at four tertiary care centers in Ankara, Türkiye, from January 2020 to December 2024: 1) Department of Physical Medicine and Rehabilitation, Ankara University Faculty of Medicine; 2) Department of Physical Medicine and Rehabilitation, Hacettepe University Faculty of Medicine; 3) Department of Physical Medicine and Rehabilitation, Gazi University Faculty of Medicine; and 4) Physical Medicine and Rehabilitation Hospital at Ankara Etlik City Hospital.

Postmenopausal women aged between 45 and 70 years who applied to the outpatient clinics of the study centers with bone mineral density (BMD) results were invited to participate in the study. They self-reported their menopausal status. Menopause was defined as the end of menstrual periods following 12 months of amenorrhea [11]. Individuals with infective, orthopedic, rheumatological, neurological, endocrinological, metabolic, or systemic diseases that might result in a decline in physical function and muscle strength were excluded. Individuals who met the participation criteria and who provided informed consent were included in the study.

The study was designed as a case-control study. The participants with osteoporosis constituted the case group, while nonosteoporotic women formed the control group. Participants who met at least one of the following criteria were diagnosed with osteoporosis: 1) T-score of −2.5 or below in the lumbar spine or femoral neck; 2) low-trauma spine or hip fracture; 3) T-score ranging from −1.0 to −2.5 and a fragility fracture of the proximal humerus, pelvis, or distal forearm; 4) prior diagnosis of osteoporosis [12]. The Discovery (Hologic, Marlborough, MA, USA), Horizon (Hologic), Stratos (Diagnostic Medical Systems, Pérols, France), and FDX Visionary (Fujifilm, Tokyo, Japan) dual-energy X-ray absorptiometry (DXA) systems were utilized to determine the BMD and T-scores of the lumbar spine (L1–L4) and femoral neck. To detect a 20% difference in sarcopenia prevalence between the nonosteoporotic [13] and osteoporotic [14] groups with α = 0.05 and 1 – β = 0.90, the necessary sample size was estimated to be 232 (116 per group).

The following assessments were conducted for all participants: participants were asked about sociodemographic features including age, weight, height, marital status, educational level, employment status, residence, style of clothing, age at menarche, age at menopause, number of pregnancies, number of live births, and duration of breastfeeding and their answers were recorded. BMI values were calculated using the formula BMI = weight/(height)^2^. Clinical risk factors for osteoporosis (previous fracture, parental hip fracture, current smoking, glucocorticoids, inflammatory rheumatic diseases, type 1 diabetes mellitus, osteogenesis imperfecta, hyperthyroidism, premature menopause, chronic malabsorption, chronic liver or kidney disease, and alcohol use) were determined [15,16]. The use of medications associated with osteoporosis [antirheumatic drugs, antineoplastic agents, antiepileptic drugs, heparin, thyroxin, antacids, and proton pump inhibitors (PPIs)] [17] and antiosteoporotic agents that were currently being used or had been used for a period of 1 year or more in the past were recorded. Weekly protein intake was assessed by recording the weekly consumption frequency and amounts of various food groups including dairy products, poultry, red meat, seafood, eggs, legumes, fresh vegetables, and grains based on previously standardized portion sizes [18]. These data were then used to calculate average daily protein intake.

The Turkish version of the 5-item SARC-F questionnaire was utilized to screen for sarcopenia risk [19,20]. It includes assessments of strength, assistance in walking, rising from a chair, climbing stairs, and falls. A score of 4 or higher suggests a sarcopenia risk [20]. For the assessment of upper limb muscle strength, grip strength was measured using a Jamar hand dynamometer (Lafayette Instrument Company, Lafayette, IN, USA) while the participant was seated, with the shoulder adducted and neutrally rotated, the elbow flexed at 90°, the forearm in neutral, and the wrist between 0 and 30° of dorsiflexion [21]. The measurement was performed three times for both sides. Participants were instructed to exert maximum effort during the assessment. For the analyses, the highest value was considered. Participants who had grip strength of less than 16 kg were diagnosed as having “probable sarcopenia” according to the criteria of the European Working Group on Sarcopenia in Older People 2 (EWGSOP2) [2]. The five-times sit-to-stand test (5T-STS) was performed to evaluate the strength of the lower limbs [2]. Participants, seated on a chair with their arms crossed over their chests, were asked to stand and sit five times consecutively and as quickly as possible. The time from the beginning of the first standing to the end of the last sitting was recorded and was utilized as a measure of lower limb strength [22]. The Turkish version of the International Physical Activity Questionnaire-Short Form (IPAQ-SF), comprising 7 items, was administered to assess physical activity level (metabolic equivalent of task (MET)-min/week) [23].

IBM SPSS Statistics 30.0 (IBM Corp., Armonk, NY, USA) was used to perform statistical analysis. Descriptive data were presented as mean ± standard deviation or median (25th–75th percentile or minimum–maximum) for metric variables according to distribution and as number (percentage) for categorical variables. The distributions of variables were analyzed with the Kolmogorov–Smirnov test. The lumbar spine and femoral neck BMD, sociodemographic features, clinical risk factors for osteoporosis, medication use, SARC-F score, 5T-STS score, grip strength, probable sarcopenia diagnosis rate, protein intake, and IPAQ-SF score were analyzed for the osteoporotic and nonosteoporotic groups using the Mann–Whitney U test, Pearson chi-square test, Fisher exact test, and independent-samples t-test. The sociodemographic features, clinical risk factors for osteoporosis, medication use, 5T-STS, grip strength, probable sarcopenia diagnosis rate, protein intake, and IPAQ-SF scores of the participants with and without SARC-F-defined sarcopenia risk were further analyzed using the Mann–Whitney U test, Pearson chi-square test, Fisher exact test, and independent-samples t-test. The Spearman correlation test was employed to explore the relationships between the BMD of the lumbar spine and femoral neck and grip strength and 5T-STS score. Multivariate logistic regression analysis was performed to identify factors affecting SARC-F-defined sarcopenia risk. Factors previously reported as potentially clinically relevant to sarcopenia [4,24–26], as well as potential confounding factors showing baseline differences between the groups in univariate comparison, were included in the model. To avoid multicollinearity, independent variables were assessed via Spearman correlation and those showing strong correlation (r > 0.70) were not included in the model simultaneously. Missing data were handled using available-case analysis for descriptive and bivariate statistics. For multivariate regression models, a complete-case analysis approach was employed. Values of p < 0.05 indicated statistical significance.

## 3. Results

The study included a total of 238 participants, with 119 participants in each group. The median (minimum–maximum) ages of the osteoporotic and nonosteoporotic groups were 58 (47–69) and 59 (46–70) years, respectively. In the osteoporotic group, the median (25th–75th percentile) BMD values of the lumbar spine [0.730 (0.695–0.769) vs. 0.941 (0.870–1.034) g/cm^2^, p < 0.001] and femoral neck [0.664 (0.608–0.751) vs. 0.832 (0.735–0.924) g/cm^2^, p < 0.001] were significantly lower than those of the nonosteoporotic group. All assessments were completed for all participants. However, there were missing results for protein intake (n = 3), 5T-STS score (n = 2), grip strength (n = 3), and IPAQ-SF (n = 1) due to registration errors.

The sociodemographic features of the participants are presented in Table 1. In the osteoporotic group, BMI (p < 0.001), the frequency of Muslim-style clothing (p = 0.022), and numbers of pregnancies (p = 0.020) and live births (p = 0.026) were significantly lower than in the nonosteoporotic group. The educational level of the osteoporotic group was significantly higher (p = 0.006) than that of the nonosteoporotic group.

Clinical risk factors for osteoporosis and the medication use of the participants are displayed in Table 2. None of the participants were diagnosed with type 1 diabetes mellitus, hypogonadism, or osteogenesis imperfecta and none were using alcohol, antineoplastic agents, or heparin. In the osteoporotic group, previous fractures (p < 0.001) and current smoking (p = 0.026) were significantly more prevalent than in the nonosteoporotic group. In the nonosteoporotic group, the use of thyroxin (p = 0.029) and PPI (p = 0.013) was significantly higher than in the osteoporotic group. In the osteoporotic group, 57 (47.9%) participants were actively using an antiosteoporotic agent [12 (10.1%) alendronate, 28 (23.5%) zoledronate, 6 (5%) ibandronate, and 11 (9.2%) denosumab] when they were recruited for the study. In the osteoporotic group, 9 (7.6%) participants were not receiving antiosteoporotic treatment at the time of their recruitment. However, these participants had used at least one agent in the past [3 (2.5%) alendronate, 1 (0.8%) zoledronate, 2 (1.7%) ibandronate, 1 (0.8%) both alendronate and ibandronate, and 2 (1.7%) more than two agents]. The remaining 53 (44.5%) participants had not used an osteoporotic agent.

Based on SARC-F scores, there was a risk of sarcopenia in 104 (44.7%) participants. According to the EWGSOP2 criteria, 35 (14.9%) participants had probable sarcopenia. The SARC-F-defined sarcopenia risk, muscle strength, frequency of probable sarcopenia, amount of protein intake, and physical activity levels of the participants are presented in Table 3. There were no significant differences regarding SARC-F-defined sarcopenia risk (p = 0.117) and frequency of probable sarcopenia (p = 0.876) between the osteoporotic and nonosteoporotic groups.

Comparisons of the participants with and without SARC-F-defined sarcopenia risk regarding sociodemographic features, clinical risk factors for osteoporosis, medication use, muscle strength, frequency of probable sarcopenia, amount of protein intake, and physical activity levels are presented in Table 4. The results of multivariate logistic regression analysis for SARC-F-defined sarcopenia risk (Nagelkerke R^2^ = 0.237) are presented in Table 5. Although educational level [odds ratio (OR): 0.366, 95% confidence interval (95% CI): 0.189–0.707, p = 0.003], PPI use (OR: 2.494, 95% CI: 1.227–5.068, p = 0.012), and physical activity level (per 100 MET-min/week; OR: 0.966, 95% CI: 0.945–0.988, p = 0.003) were independent predictors of SARC-F-defined sarcopenia risk, osteoporosis (OR: 1.560, 95% CI: 0.820–2.967, p = 0.176) was not.

In our study sample, there was no significant correlation of grip strength with lumbar spine BMD (r = −0.048, p = 0.464, n = 233) or femoral neck BMD (r = 0.127, p = 0.052, n = 235). Similarly, lower extremity strength estimated by the 5T-STS test was not significantly correlated with lumbar spine BMD (r = −0.001, p = 0.989, n = 234) or femoral neck BMD (r = −0.097, p = 0.137, n = 236).

## 4. Discussion

The results of this study revealed that the frequency of sarcopenia risk, as assessed via the SARC-F, was 48.7% in the osteoporotic group and 38.7% in the nonosteoporotic group among postmenopausal women aged ≤70 years. However, no statistically significant difference regarding sarcopenia risk was detected between the two groups. Furthermore, lower educational status and physical activity levels, as well as PPI use, were independently associated with an increase in SARC-F-defined sarcopenia risk.

Sarcopenia is a prevalent disease of the skeletal muscle [2]. Two metaanalyses including women aged 60 years and older in the community reported the sarcopenia prevalence as 9% and 10%, respectively [6,27]. This rate was further elevated in individuals in nursing homes or hospitals [27]. Sarcopenia is not only a disease of the elderly. More than one-tenth of young adults were also reported to suffer from sarcopenia [28]. Studies investigating the prevalence of sarcopenia in middle-aged postmenopausal women are scarce, however. In a study using a combination of the EWGSOP and Asian Working Group for Sarcopenia criteria, it was reported that 3% of postmenopausal women aged 45–65 years had sarcopenia [24]. In another study, according to the EWGSOP2 criteria, 36% and 31% of postmenopausal women aged 54–80 years were diagnosed with probable sarcopenia and sarcopenia, respectively [29]. In our study, 14.9% of the participants were classified as having probable sarcopenia. Variations in the selected populations and sarcopenia criteria among studies may lead to reports of disparate outcomes.

We assessed sarcopenia risk using the SARC-F, a tool that primarily focuses on functional activities related to the lower extremities [20]. To determine probable sarcopenia, we performed measurements of grip strength, which facilitates assessment of the upper extremities, contrary to the SARC-F. In our study, probable sarcopenia was more often detected in participants with SARC-F-defined sarcopenia risk than in those without sarcopenia risk. Both the SARC-F and measurement of grip strength yielded comparable findings during the assessment of sarcopenia despite evaluating distinct body parts. According to the EWGSOP2 criteria, following an initial evaluation with the SARC-F and muscle strength testing, the diagnosis of sarcopenia must be confirmed through the evaluation of muscle mass [2]. Therefore, our findings represent sarcopenia risk at the screening level rather than a confirmed clinical diagnosis, since muscle mass was not objectively measured. The Global Leadership Initiative in Sarcopenia proposed muscle mass, muscle strength, and muscle-specific strength as components of the definition of sarcopenia. Impaired physical performance, inability to perform activities of daily living, mobility limitations, falls, fractures, hospitalization, and mortality were identified as outcomes of sarcopenia [30]. Accordingly, given its specific items, the SARC-F provides an indirect assessment of both sarcopenia and its outcomes [20]. Furthermore, previous studies reported that the SARC-F exhibits moderate to high sensitivity and specificity for sarcopenia screening [31,32]. Therefore, our results based on the SARC-F remain valuable for determining sarcopenia risk, although direct measurement of muscle mass was not performed.

In our study, we found no statistically significant increased sarcopenia risk, as assessed via the SARC-F, in postmenopausal osteoporotic women aged ≤70 years compared to the nonosteoporotic control group, although the frequency was higher in the osteoporotic group. Additionally, there were no differences in grip and lower limb strength or the frequency of probable sarcopenia between these groups. Furthermore, the BMD of the lumbar spine and femoral neck was not correlated with grip and lower limb strength. To adjust for potential confounding factors arising from baseline differences in sociodemographic features, clinical risk factors, and medication use, multivariate regression analysis was conducted. Accordingly, postmenopausal osteoporosis was not an independent predictor of SARC-F-defined sarcopenia risk, consistent with our initial findings. However, the possibility of residual confounding cannot be completely excluded despite this adjustment. In the elderly, sarcopenia and osteoporosis are related diseases [8]. Sarcopenia results from mitochondrial dysfunction, intramuscular fat infiltration, and neuromuscular junction deterioration associated with aging [33], and osteoporosis arises from bone marrow alterations due to senility [34]. Menopausal transition was also reported to result in reductions in both skeletal muscle mass and BMD [26,35]. However, the main mechanisms in postmenopausal women are different than those in the elderly. A decrease in estrogen levels following menopause leads to postmenopausal osteoporosis [36]. Skeletal muscle loss may result from crosstalk between muscle and bone tissue via mechanical, paracrine, and endocrine pathways and the direct effects of estrogen on muscle tissue [7,37]. Although there is strong evidence regarding the impact of estrogen on bone tissue, reliable evidence for muscle tissue is scarce. The efficacy of estrogen in the treatment of sarcopenia is uncertain [37,38]. On the other hand, in one study, the prevalence of sarcopenia was demonstrated to be lower in postmenopausal women receiving hormone replacement therapy for longer durations, especially those aged <65 years and with BMI of <25 kg/m^2^ [39]. A previous study including postmenopausal women with a mean age of 60.5 years, constituting a demographic highly similar to that of our study population, supported our findings and reported that there was no difference in sarcopenia prevalence between the groups with and without osteoporosis [40]. Furthermore, while another study observed lower skeletal muscle mass in osteoporotic postmenopausal women younger than 60 years compared to their nonosteoporotic counterparts, there was no statistically significant difference in the prevalence of sarcopenia between the groups [41]. Nevertheless, in some studies involving postmenopausal women, sarcopenia and its components were observed to be associated with osteoporosis; however, the participants in those studies were older than 65 years [9,42]. Consequently, while estrogen undoubtedly exerts favorable effects on both bone and muscle tissues, the decline in its levels following menopause may affect these tissues at different rates. Muscle deterioration may not have been as pronounced as bone demineralization in our relatively younger postmenopausal population compared to the elderly. Therefore, an association between SARC-F-defined sarcopenia risk and osteoporosis may not have been observed in our study.

Nearly half of our entire study population had SARC-F-defined sarcopenia risk. It is crucial to identify the factors associated with sarcopenia in postmenopausal women because of its adverse outcomes, such as falls, fractures, physical disability, and mortality. The prevalence of sarcopenia among the elderly was reported to increase with advancing age [4]. Although our initial analysis indicated that the participants with SARC-F-defined sarcopenia risk were older than those without such risk, multivariate regression analysis demonstrated that age was not an independent predictor of this risk. Likewise, another study including postmenopausal women aged 45–65 years revealed that age was not a risk factor for low muscle mass [24]. A lower BMI was reported to be related to lower muscle mass and strength, as well as sarcopenia, in postmenopausal women [24,43]. In particular, BMI of ≤20 kg/m^2^ was independently associated with lower muscle mass in postmenopausal women. A BMI at that level could be a sign of malnutrition [24]. Insufficient consumption of nutritional elements hinders the maintenance of muscle mass and strength [44]. In postmenopausal women, adherence to the Mediterranean diet was related to lean mass and muscle strength [45], and protein consumption was positively associated with muscle mass [25]. In our study, there was no apparent difference in BMI values between participants with and without SARC-F-defined sarcopenia risk, and BMI was not an independent predictor of this risk. Nevertheless, the mean BMI of both groups was classified as overweight [46]. The median protein intake of participants with SARC-F-defined sarcopenia risk was lower than that of the other group, although the difference was not statistically significant. Correspondingly, protein intake did not emerge as an independent predictor of this risk in the multivariate regression analysis. Nevertheless, the amount of protein intake of the entire study population was lower than the daily protein requirement of adults reported by the World Health Organization [47]. In studies including postmenopausal women, higher levels of physical activity were found to be associated with greater muscle mass and lower sarcopenia prevalence [25,26,39]. Similarly, participants with SARC-F-defined sarcopenia risk in our study had a lower level of physical activity compared to the other group. This finding was further supported by multivariate regression analysis. In previous studies, resistance exercise was demonstrated to increase muscle mass and strength in postmenopausal women [48], and protein supplementation, when paired with resistance exercise, amplified this impact in both younger and older adults [49,50]. According to our findings, higher educational status was independently associated with a lower SARC-F-defined sarcopenia risk. Consistent with this, in one study involving postmenopausal women, higher educational status was reported to be linked to greater handgrip strength [51]. Previous studies demonstrated that older adults with lower educational status exhibited poor knowledge of sarcopenia, whereas an increase in knowledge led to improved attitudes and practices for preventing sarcopenia [52,53]. This observation is likely applicable to postmenopausal women as well.

PPI use emerged as an independent predictor of SARC-F-defined sarcopenia risk in our study. Parallel to our findings, a previous study of adults found that the use of PPIs, especially esomeprazole and lansoprazole, and longer duration of use were associated with reduced muscle mass. This may be attributed to the decreased absorption of amino acids and key nutrients, such as magnesium, vitamin B12, and vitamin D, due to suppressed gastric acidity, as well as chronic inflammation resulting from changes in the gut microbiota and disruption of the intestinal barrier [54]. The studies mentioned above [24–26,39,43,45,48,51] included postmenopausal women with ages similar to those of our study participants or younger. Considering the results of these studies and our own, sarcopenia and SARC-F-defined sarcopenia risk may be associated with malnutrition, inadequate protein intake, lower educational status, PPI use, and low levels of physical activity in postmenopausal women aged 70 years and younger.

This study has some notable strengths. It expands upon the current literature by providing novel insight into the relationship between osteoporosis and SARC-F-defined sarcopenia risk, specifically in postmenopausal women aged 70 years and younger. With that goal, it utilized widely accepted assessment methodologies for osteoporosis and sarcopenia. Furthermore, it had a multicenter design, increasing the generalizability of the results.

This study also has several limitations. First, sarcopenia was not directly evaluated as measurements of skeletal muscle mass were not performed. Nonetheless, assessments of SARC-F-defined sarcopenia risk and probable sarcopenia, which are preliminary steps in diagnosing confirmed sarcopenia following the EWGSOP2 algorithm [2], were conducted. Despite the favorable performance of the SARC-F for screening, this lack of direct measurement may have limited the ability to make definitive inferences regarding the true prevalence of sarcopenia in our study population, as well as the sarcopenia risk in the community based on our results. Second, this was a multicenter study, and DXA systems from different manufacturers were utilized by different centers to determine BMD and T-scores. Although routine calibrations were performed at each center, the measurements from different DXA systems may not have been consistent [55], possibly resulting in minor discrepancies in the allocation of some participants to groups. Third, the amount of protein intake was assessed by a nonvalidated questionnaire that inquired about the frequency and quantity of consumption of various food groups. Diverse interpretations of portion sizes by the participants may have influenced outcomes regarding protein intake. Fourth, objective measurements of physical activity were not feasible due to time and equipment constraints. Therefore, the IPAQ-SF, which relies on self-reporting and is thus subject to recall bias, was utilized. Nevertheless, the IPAQ-SF is a validated tool for assessing physical activity [23]. Finally, the study was conducted in four tertiary care centers where patients apply as the last step for healthcare. While the multicenter design enhanced the generalizability of the results, performing the study solely in tertiary centers may have detracted from the generalizability. Future studies should focus on the relationship between confirmed sarcopenia and postmenopausal osteoporosis in women aged 70 years and younger.

In conclusion, compared to a nonosteoporotic control group, no increase in SARC-F-defined sarcopenia risk was observed in women with postmenopausal osteoporosis aged 70 years and younger. Although lower educational status, PPI use, and low physical activity levels were independently associated with an increased SARC-F-defined sarcopenia risk, osteoporosis was not.

## Figures and Tables

**Table 1:** Sociodemographic features of the participants.

	Total (n = 238)	Osteoporotic (n = 119)	Nonosteoporotic (n = 119)	p
Age (years)	59 (54–63)	58 (55–63)	59 (54–64)	0.933^a^
Body mass index (kg/m^2^)	29.14 ± 5.33	27.33 ± 5.06	30.96 ± 4.98	**< 0.001** b
Marital status (married)	184 (77.3)	89 (74.8)	95 (79.8)	0.353^c^
Educational level (>5 years)	97 (40.8)	59 (49.6)	38 (31.9)	**0.006** c
Employment status (working)	47 (19.7)	18 (15.1)	29 (24.4)	0.073^c^
Residence (urban)	224 (94.1)	115 (96.6)	109 (91.6)	0.098^c^
Dressing (with Muslim-style clothing)	151 (63.4)	67 (56.3)	84 (70.6)	**0.022** c
Age at menarche (years)	14 (12–14)	14 (12–15)	13 (12–14)	0.931^a^
Age at menopause (years)	47 (42–50)	46 (41–50)	47 (43–50)	0.495^a^
Number of pregnancies	3 (2–4)	3 (2–4)	4 (2–4)	**0.020** a
Number of live births	3 (2–3.25)	2 (2–3)	3 (2–4)	**0.026** a
Duration of breastfeeding (months)	29 (11.75–54)	24 (11–54)	32 (12–54)	0.473^a^

Values are presented as median (25th–75th percentile), mean ± standard deviation, or number (percentage).

a Mann–Whitney U test;

b independent-samples t-test;

c Pearson chi-square test.

Bolded p-values indicate statistical significance.

**Table 2:** Clinical risk factors for osteoporosis and medication use of the participants.

	Total (n = 238)	Osteoporotic (n = 119)	Nonosteoporotic (n = 119)	p
**Clinical risk factors for osteoporosis**				
Previous fracture	22 (9.2)	22 (18.5)	0 (0)	**< 0.001** a
Parental hip fracture	35 (14.7)	19 (16)	16 (13.4)	0.583^a^
Current smoking	41 (17.2)	27 (22.7)	14 (11.8)	**0.026** a
Glucocorticoids	21 (8.8)	10 (8.4)	11 (9.2)	0.819^a^
Inflammatory rheumatic disease	19 (8)	6 (5)	13 (10.9)	0.094^a^
Hyperthyroidism	1 (0.4)	1 (0.8)	0 (0)	1.000^b^
Premature menopause	76 (31.9)	43 (36.1)	33 (27.7)	0.164^a^
Chronic malabsorption	1 (0.4)	1 (0.8)	0 (0)	1.000^b^
Chronic liver or kidney disease	11 (4.6)	4 (3.4)	7 (5.9)	0.354^a^
**Medication use**				
Antirheumatic drugs	15 (6.3)	5 (4.2)	10 (8.4)	0.182^a^
Antiepileptic drugs	7 (2.9)	1 (0.8)	6 (5)	0.119^b^
Thyroxin	43 (18.1)	15 (12.6)	28 (23.5)	**0.029** a
Antacids	11 (4.6)	5 (4.2)	6 (5)	0.758^a^
Proton pump inhibitors	54 (22.7)	19 (16)	35 (29.4)	**0.013** a

Values are shown as number (percentage).

a Pearson chi-square test;

b Fisher exact test. Bolded p-values indicate statistical significance.

**Table 3:** Comparison of SARC-F-defined sarcopenia risk, muscle strength, probable sarcopenia, protein intake, and physical activity levels between the groups.

	Total	n	Osteoporotic	n	Nonosteoporotic	p
Sarcopenia risk (SARC-F ≥ 4)	104 (43.7)	119	58 (48.7)	119	46 (38.7)	0.117^a^
5T-STS (s)	13.93 (11.74–16)	118	13.90 (11.74–16)	118	13.96 (11.72–15.90)	0.986^b^
Grip strength (kg)	22 (18–26)	117	23 (18.15–27)	118	22 (17.75–26)	0.329^b^
Probable sarcopenia (grip strength < 16 kg)	35 (14.9)	117	17 (14.5)	118	18 (15.3)	0.876^a^
Protein intake/body weight (g/kg/day)	0.56 (0.42–0.72)	116	0.55 (0.42–0.72)	119	0.56 (0.42–0.71)	0.972^b^
IPAQ-SF (MET-min/week)	809 (297–2079)	118	907.5 (379.5–1782)	119	792 (248–2226)	0.983^b^

5 T-STS: Five-times sit-to-stand test; IPAQ-SF: International Physical Activity Questionnaire-Short Form; MET: metabolic equivalent of task. Values are shown as median (25th–75th percentile), mean ± standard deviation, or number (percentage).

a Pearson chi-square test;

b Mann–Whitney U test.

Bolded p-values indicate statistical significance.

**Table 4:** Comparison of participants with and without SARC-F-defined sarcopenia risk.

	Sarcopenia risk (SARC-F ≥ 4)				p
	n	Yes	n	No	
**Sociodemographic features**					
Age (years)	104	61 (56–64)	134	58 (54–62)	**0.002** a
Body mass index (kg/m^2^)	104	29.44 ± 5.33	134	28.91 ± 5.33	0.447^b^
**Clinical risk factors for osteoporosis**					
Previous fracture	104	14 (13.5)	134	8 (6)	**0.048** c
Parental hip fracture	104	15 (14.4)	134	20 (14.9)	0.914^c^
Current smoking	104	21 (20.2)	134	20 (14.9)	0.286^c^
Glucocorticoids	104	12 (11.5)	134	9 (6.7)	0.193^c^
Inflammatory rheumatic disease	104	10 (9.6)	134	9 (6.7)	0.413^c^
Hyperthyroidism	104	1 (1)	134	0 (0)	0.437^d^
Premature menopause	104	38 (36.5)	134	38 (28.4)	0.179^c^
Chronic malabsorption	104	0 (0)	134	1 (0.7)	1.000^d^
Chronic liver or kidney disease	104	7 (6.7)	134	4 (3)	0.218^d^
**Medication use**					
Antirheumatic drugs	104	8 (7.7)	134	7 (5.2)	0.437^c^
Antiepileptic drugs	104	5 (4.8)	134	2 (1.5)	0.245^d^
Thyroxin	104	19 (18.3)	134	24 (17.9)	0.943^c^
Antacids	104	8 (7.7)	134	3 (2.2)	0.062^d^
Proton pump inhibitors	104	33 (31.7)	134	21 (15.7)	**0.003** c
**Muscle strength, probable sarcopenia, protein intake, and physical activity levels**					
5T-STS (s)	103	15 (12.94–17.56)	133	13 (11.08–14.89)	**< 0.001** a
Grip strength (kg)	102	19 (15.43–24.10)	133	24 (21–28)	**< 0.001** a
Probable sarcopenia (grip strength < 16 kg)	102	27 (26.5)	133	8 (6)	**< 0.001** c
Protein intake/body weight (g/kg/day)	103	0.52 (0.40–0.71)	132	0.57 (0.44–0.73)	0.112^a^
IPAQ-SF (MET-min/week)	104	544.5 (74.25–1386)	133	1188 (594–2305.5)	**< 0.001** a

5 T-STS: Five-times sit-to-stand test; IPAQ-SF: International Physical Activity Questionnaire-Short Form; MET: metabolic equivalent of task. Values are shown as median (25th–75th percentile), mean ± standard deviation, or number (percentage).

a Mann–Whitney U test;

b independent-samples t-test,

c Pearson chi-square test;

d Fisher exact test.

Bolded p-values indicate statistical significance.

**Table 5:** Multivariate logistic regression analysis for SARC-F-defined sarcopenia risk.

Variables	OR (95% CI)	p
Age (years)	1.019 (0.986–1.054)	0.268
Body mass index (kg/m^2^)	0.969 (0.914–1.026)	0.280
Educational level (>5 years)	0.366 (0.189–0.707)	**0.003**
Dressing (with Muslim-style clothing)	0.781 (0.389–1.565)	0.486
Number of live births	1.180 (0.958–1.453)	0.119
Previous fracture	2.159 (0.718–6.498)	0.171
Current smoking	1.171 (0.528–2.598)	0.697
Thyroxin	0.917 (0.426–1.972)	0.824
Proton pump inhibitors	2.494 (1.227–5.068)	**0.012**
Protein intake/body weight (g/kg/day)	0.519 (0.165–1.637)	0.263
IPAQ-SF (MET-min/week)*	0.966 (0.945–0.988)	**0.003**
Osteoporosis	1.560 (0.820–2.967)	0.176

CI: Confidence interval; IPAQ-SF: International Physical Activity Questionnaire-Short Form; MET: Metabolic equivalent of task; OR: odds ratio;

* OR represents the change in risk for every 100 MET-min/week increase in physical activity.

Bolded p-values indicate statistical significance.

## Data Availability

The data that support the findings of this study are available from the corresponding author upon reasonable request.
